# Complete genomes from a xenic *Dolichospermum flosaquae* FBCC-A233 culture reveal genome-inferred metabolic asymmetry with associated bacteria

**DOI:** 10.1128/spectrum.01014-26

**Published:** 2026-07-31

**Authors:** Jina Kim, Hyaekang Kim, Jaeduk Goh, Seung Won Nam, Eu Jin Chung, Seungmin Shin, Yejin Park, Youngmin Han, Ji-Eun Kim, Woori Kwak

**Affiliations:** 1 Department of Agricultural Biotechnology and Research Institute of Agriculture and Life Sciences, Seoul National University 539783, Gwanak-gu, Seoul, Republic of Korea; 2 Bio-resources Bank Division, Nakdonggang National Institute of Biological Resources 443035, Sangju, Republic of Korea; 3 Fungi Research Division, Biological Resources Research Department, Nakdonggang National Institute of Biological Resources 443035, Sangju, Republic of Korea; 4 Using Technology Development Department, Nakdonggang National Institute of Biological Resources 443035, Sangju, South Korea; 5 Division of Cardiology, Emory University School of Medicine 12239, Atlanta, Georgia, USA; 6 Department of Medical and Biological Sciences, The Catholic University of Korea 26713, Bucheon, Republic of Korea; 7 Department of Biotechnology, The Catholic University of Korea 26713, Bucheon, Republic of Korea; Connecticut Agricultural Experiment Station, New Haven, Connecticut, USA

**Keywords:** *Dolichospermum flos-aquae*, phycosphere, metagenomics, complete genomes

## Abstract

Cyanobacteria form phycosphere communities with associated bacteria, but genome-resolved resources are needed to formulate testable hypotheses about their metabolic interactions. Here, we reconstructed three complete circular genomes from a unialgal xenic culture, including *Dolichospermum flosaquae* FBCC-A233 and two associated alphaproteobacterial genomes assigned to *Sphingorhabdus* sp. and *Brevundimonas* sp. Genome-wide read mapping and genome-quality assessment supported the three recovered genomes as high-quality circular reconstructions. Comparative genome analysis placed the cyanobacterial genome within the *Dolichospermum flosaquae* species cluster under the GTDB framework, while the associated bacterial genomes represented *Sphingorhabdus* sp. and a putative undescribed *Brevundimonas* species-level lineage. Genome architecture analysis indicated reduced genome size and gene content in *Brevundimonas* relative to genus-level references although additional metrics did not support a strong conclusion of classical genome streamlining. Selected KEGG module and KO-level reconstructions indicated genome-inferred metabolic asymmetries across the consortium. FBCC-A233 encoded photosynthesis- and nitrogen-related modules and a BioU-mediated *de novo* biotin biosynthesis route, whereas the associated bacteria lacked complete *de novo* biotin biosynthesis but retained biotin-dependent carboxylase genes. FBCC-A233 also encoded extensive anaerobic corrinoid biosynthesis potential; however, canonical DMB-containing cobalamin completion, cobamide identity, and complete transporter systems were not resolved. Together, these complete genomes provide a genome-resolved resource for investigating genome-inferred metabolic differentiation and ecological interactions in cyanobacteria-associated bacterial consortia.

**IMPORTANCE:**

Phycosphere interactions between cyanobacteria and associated bacteria can shape aquatic microbial communities, but many proposed interactions remain difficult to evaluate without genome-resolved resources. This study provides three complete circular genomes from a unialgal xenic *Dolichospermum flosaquae* culture, capturing the cyanobacterium and two co-maintained bacterial associates. Our analysis identifies genome-inferred metabolic asymmetries, particularly in biotin- and cobamide-related pathways. *D. flosaquae* FBCC-A233 encoded candidate *de novo* biotin and corrinoid biosynthesis capacity, whereas the associated bacteria lacked complete *de novo* pathways but retained cofactor-dependent enzymes. These findings nominate cofactor-related dependencies as experimentally testable hypotheses while emphasizing unresolved uptake, export, cobamide identity, and growth-dependence mechanisms. The complete genomes and KO-level reconstructions generated here provide a resource for future studies of cyanobacteria-associated consortia.

## INTRODUCTION

Cyanobacteria are photosynthetic microorganisms that play pivotal roles in aquatic ecosystems. As primary producers, they contribute to organic matter production as well as carbon and nitrogen cycling (1). Some cyanobacteria can persist under nutrient-limited conditions through biological nitrogen fixation (2). Under favorable environmental conditions, cyanobacteria can form blooms that alter ecosystem functioning, leading to hypoxia driven by oxygen depletion and the release of bioactive or toxic metabolites (3, 4). These phenomena have significant ecological, economic, and public health impacts. Accordingly, understanding the ecological processes that support cyanobacterial persistence and bloom formation remains a priority in aquatic systems.

*Dolichospermum* is a filamentous cyanobacterial genus characterized by cellular differentiation, including the formation of heterocysts and akinetes (5). Heterocysts support nitrogen fixation, whereas akinetes promote survival under unfavorable conditions (6, 7). Filament integrity also permits intercellular connectivity and metabolite exchange, enabling coordinated metabolism within filaments (8). This functional plasticity allows *Dolichospermum* to respond to changes in temperature, nutrient availability, and light conditions, potentially contributing to seasonal blooms and long-term ecological succession (9). Previous studies have reported that nitrogen-fixing cyanobacteria can release reduced nitrogen compounds and photosynthate-derived organic matter that support associated bacteria and that associated bacteria may provide metabolites beneficial to cyanobacteria, such as vitamins, organic growth factors, or trace nutrients including iron (10, 11). The phycosphere, the microenvironment surrounding cyanobacterial cells and filaments, provides a microscale setting in which such interactions may occur (12, 13). In freshwater phycosphere communities, bacterial taxa frequently reported as dominant or recurrent associates include *Brevundimonas*, *Sphingomonas*, and members of the *Flavobacteriia*, suggesting that cyanobacteria-associated bacterial assemblages may contribute to nutrient turnover in aquatic environments (14–16).

However, many studies of cyanobacteria-associated communities have relied on 16S rRNA gene profiling or incomplete metagenome-assembled genomes (MAGs), thereby limiting genome-scale inference of metabolic interactions (17). In particular, although *Dolichospermum flos-aquae* genome assemblies are available in public databases, complete circular genome sequences remain scarce in public databases. To address this gap, we reconstructed three complete circular genomes from a unialgal xenic culture. These included *D. flosaquae* FBCC-A233 and two associated bacteria assigned to *Sphingorhabdus* sp. and *Brevundimonas* sp. We used these genomes to establish taxonomic placement and to infer genome-encoded functional potential within the consortium, with particular attention to cofactor-related pathways. Because cofactor exchange has been implicated in microbial interactions in other cyanobacteria-associated systems, we examined biotin- and cobamide-related functions, including the distribution of biotin biosynthesis genes and methionine synthase isoforms, as candidate indicators of potential cofactor dependence (18–20). Together, this study provides a genome-resolved framework for generating experimentally testable hypotheses about metabolic coupling and ecological differentiation within the *Dolichospermum*-associated bacterial consortia.

## MATERIALS AND METHODS

### Sample information

The FBCC-A233 culture was provided by the Freshwater Bioresources Culture Collection (FBCC), Nakdonggang National Institute of Biological Resources (NNIBR), as a unialgal xenic culture. The original field sample was collected from Inpyeong Reservoir, 321 Inpyeong-ri, Taean-eup, Taean-gun, Chungcheongnam-do, 32138, Republic of Korea (36°45′52.3″ N, 126°20′53.8″ E) in April 2019, and FBCC-A233 strain was established by single-filament isolation. The culture has been maintained in Blue-Green Medium (BG11) at 20°C under a 16:8 h light:dark cycle with a light intensity of approximately 3,000 lux and preserved by serial subculture at approximately 10-day intervals, with single-filament isolation performed during subculture.

### DNA extraction and library preparation

Genomic DNA was extracted using the Mag-Bind Universal Pathogen Kit (Omega Bio-Tek, Norcross, GA, USA). DNA purity was assessed with a NanoDrop 2000 spectrophotometer, and DNA concentration was quantified using a Qubit 4 fluorometer with the dsDNA High Sensitivity Assay Kit (Thermo Fisher Scientific, Waltham, MA, USA). A long-read sequencing library was prepared with the SQK-LSK114 ligation sequencing kit and loaded onto a Flongle R10.4.1 flow cell (FLO-FLG114) using the Oxford Nanopore Technologies platform (Oxford, UK). For short-read polishing, an Illumina library was prepared with the TruSeq Nano DNA kit and sequenced on a NovaSeq 6000 system (Illumina, San Diego, CA, USA). The Illumina short-read data set comprised 27,535,343 paired-end read pairs, corresponding to 55,070,686 reads and approximately 8.32 Gbp of total sequence yield. Illumina reads were 2 × 151 bp, with a read N50 of 151 bp. FastQC analysis indicated Sanger/Illumina 1.9 encoding with Phred+33 quality scores, approximately 50% GC content, and no sequences flagged as poor quality. Nanopore sequencing generated approximately 174.23 thousand reads, with an estimated total yield of 862.81 Mb and an estimated read N50 of 8.4 kb.

### Genome assembly, evaluation, and gene annotation

Microbial community profiles were inferred from short-read data using MetaPhlAn 4 v4.2.2 (21). Long-read basecalling was performed using Dorado v1.0.2 (https://github.com/nanoporetech/dorado, accessed on 20 July 2025) with the --trim-adapters option and the super-accuracy v5 model. Draft genomes were assembled with Flye v2.9-b1768 (22) using the --meta option. Candidate complete circular genome sequences were selected from the Flye assembly output based on three criteria: (i) megabase-scale contig size consistent with bacterial chromosomes, (ii) positive circularization status in the Flye assembly information, and (iii) absence of repeat-contig flags. Three contigs satisfied these criteria and were retained as complete circular genome sequences for downstream polishing and analysis. The retained circular contigs were subsequently polished with Medaka v2.1.1 (https://github.com/nanoporetech/medaka, accessed on 28 August 2025). To improve assembly accuracy, an additional polishing step was conducted using short-read data. Short reads trimmed with Trimmomatic v0.39 (23) were mapped to the draft assemblies using BWA-MEM2 v2.3 (24), and alignments were processed with Samtools v1.19.2 (25). Final consensus sequences for the three complete genomes were obtained by short-read polishing with Polypolish v0.6.1 (26). After final polishing, the same short-read mapping workflow was repeated against the final polished genome sequences to complement the marker-based community profile and evaluate genome-wide support for the recovered contigs. For each recovered genome, mapped read counts, mapping proportions, mean depth, and breadth of coverage at ≥1× and ≥5× were calculated from the final alignment files as genome-wide coverage-support metrics. Genomic relatedness of each complete genome was first assessed by Tetra Correlation Search (TCS) against the JSpeciesWS reference database (27). Genome completeness and contamination were assessed using BUSCO v6.0.0 (nostocales_odb12 and alphaproteobacteria_odb12) (28) and CheckM2 v1.1.0 (29). Structural and functional genome annotation was carried out with Prokka v1.14.6 (30).

### Species identification

Species identification employed a hierarchical genomic approach. Detailed taxonomic classification was conducted using GTDB-Tk v2.5.2 with the r226 reference data set (31, 32). To construct reference genome sets for taxonomic comparison, genomes were retrieved from the NCBI RefSeq Genome database (33). Because the available references included assemblies ranging from complete genomes to scaffold/contig-level assemblies, all reference genomes were first evaluated using CheckM2 v1.1.0. Genomes with >90% completeness and <5% contamination were retained. This quality-control step retained 89 of 91 ADA clade (*Anabaena*, *Dolichospermum*, and *Aphanizomenon*) genomes, 62 of 71 *Sphingorhabdus* genomes, and 416 of 445 *Brevundimonas* genomes. FastANI v1.33 (34) was then applied to the QC-filtered reference genomes to identify close reference genomes for pairwise genomic relatedness analysis, retaining only genomes with ANI >80% and alignment fraction >60%. The final ANIb/dDDH comparison set comprised 54 ADA genomes, 14 *Sphingorhabdus* genomes, and 51 *Brevundimonas* genomes. The three genomes reconstructed from the metagenome were then compared against this curated reference set using the BLAST+-based ANI (ANIb) function of JSpeciesWS (27) and analyzed with the Genome-to-Genome Distance Calculator (GGDC), with digital DNA-DNA hybridization (dDDH) values estimated based on formula 2 (35).

### Genome architecture and genome-inferred metabolic pathway analysis

Genome architecture and metabolic pathway analyses were conducted using a unified annotation framework based on Prokka gene prediction and eggNOG-mapper v2.1.13 (36) functional assignment. For genome architecture analysis, the full QC-filtered genus-level reference sets were used (62 *Sphingorhabdus* and 416 *Brevundimonas* genomes). These reference genomes and the reconstructed genomes were processed using the same Prokka workflow to ensure metric comparability. Genome architecture metrics were derived from assembly FASTA sequences and Prokka GFF features. For each bacterial associate, query values were positioned within genus-level reference distributions and summarized as percentile ranks based on the empirical cumulative distribution function. Coding density was calculated as the total length of merged CDS intervals divided by assembly size. Intergenic-region lengths were calculated as internal gaps between adjacent merged annotated features. For draft or linear reference assemblies, terminal gaps at contig ends were excluded, whereas the origin-spanning interval was included for circular query genomes. Median intergenic-region length was used as the primary intergenic metric to reduce sensitivity to long outlier intervals. Prokka-derived pseudogene candidates were identified from pseudogene-related annotations and reported as candidate counts. IS/transposase-related CDSs were identified from Prokka product annotations containing insertion sequence or transposase-related terms and normalized per Mb. Sensitivity analyses excluding high-N reference assemblies did not materially alter the percentile-based interpretation of coding density or median intergenic-region length. For selected module-level pathway completeness analysis, protein sequences predicted from the three reconstructed genomes were annotated using the KofamKOALA web server (37). KO assignments meeting the KofamKOALA default adaptive score threshold were retained, and a non-redundant KO list was generated for each genome. These genome-specific KO lists were used as input for kegg-pathway-completeness v1.4.3 (38) to calculate weighted KEGG module completeness across the three genomes. The resulting module-level outputs, including module accession, completeness, pathway name, pathway class, matching KOs, and missing KOs, were compiled as genome-specific sheets in Table S3. Selected modules related to photosynthesis and nitrogen metabolism, carbon and respiratory metabolism, sulfolipid/sulfoquinovose metabolism, and cofactor-related pathways were visualized in R as a heatmap. KO-level maps for biotin and cobamide/corrinoid-related pathways were generated from the same KofamKOALA-derived KO profiles and referenced the KEGG Mapper (39).

## RESULTS

### Community profiling and recovery of three complete circular genomes from a unialgal xenic culture

**Fig 1:**
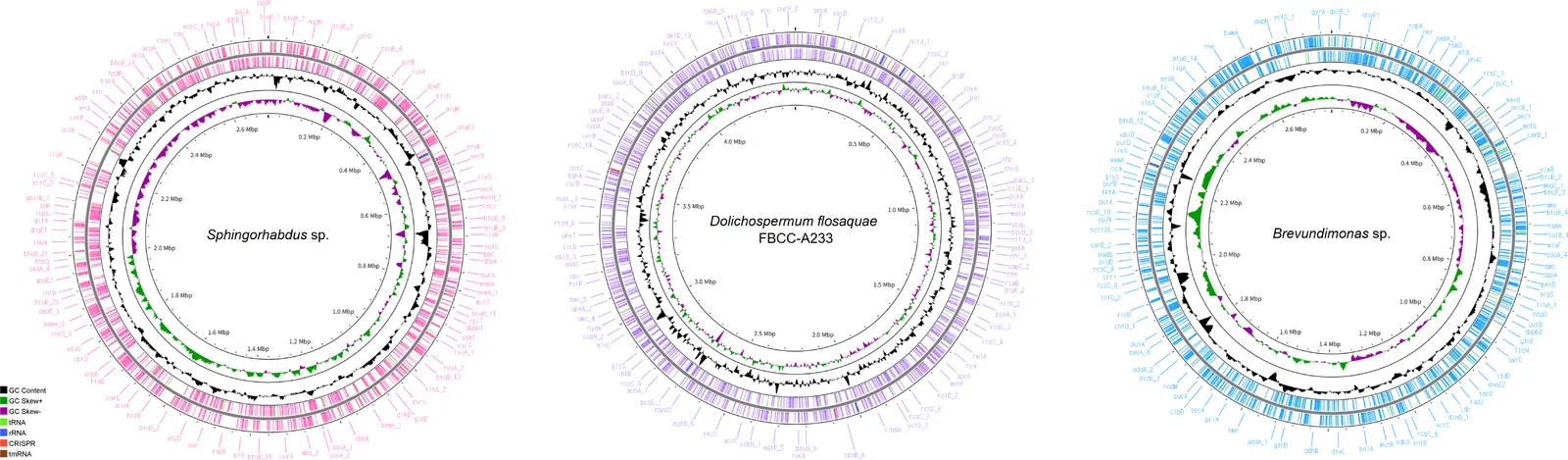
Circular genome maps of the three complete genomes (*Sphingorhabdus* sp., *D. flosaquae* FBCC-A233, and *Brevundimonas* sp.). The outer rings display predicted coding sequences on the forward and reverse strands. Inner tracks illustrate genome-wide compositional features, including GC content and positive and negative GC skew, plotted relative to genomic coordinates. Additional feature tracks indicate tRNA, rRNA, CRISPR arrays, and tmRNA loci. Tick marks denote genome positions in megabases.

Because FBCC-A233 was maintained as a unialgal xenic culture, we first characterized the co-maintained microbial community using marker-based profiling (Table 1). Although a substantial fraction of reads remained unclassified at the applied threshold (55.57%), the dominant classified fraction was assigned to a cyanobacterial taxon identified as *Aphanizomenon flos-aquae* (37.82%), together with several lower-abundance bacterial lineages, including taxa such as GGB132637 SGB181770 (2.57%) and *Pseudomonas* SGB60145 (2.38%). Because the marker-based profile indicated a mixed cyanobacterial-bacterial community but left a large fraction of reads unclassified, we applied a metagenome-resolved assembly strategy combining long-read assembly and short-read polishing. This approach yielded three circular contigs, which were subsequently refined into three complete circular genomes (Fig. 1; Table 2). These results showed that multiple community members from the unialgal xenic culture could be resolved at the genome level. As an initial taxonomic screen, we compared the three genomes with reference genomes using a composition-based similarity search. The closest matches were *Aphanizomenon flos-aquae* LD13, *Sphingorhabdus wooponensis* 03SU3-P, and *Brevundimonas subvibrioides* ATCC 15264, with *Z*-scores of 0.99924, 0.96743, and 0.94511, respectively. These results suggested that the recovered genomes represented one cyanobacterial genome and two alphaproteobacterial genomes affiliated with *Sphingorhabdus* and *Brevundimonas*.

**TABLE 1:** Microbial community composition of the non-axenic culture, determined by MetaPhlAn 4 analysis^*a*^

Clade name	Relative abundance (%)	Coverage	Estimated reads
UNCLASSIFIED	55.57449	-	30,605,193
*Aphanizomenon flos-aquae*	37.82080748	4.655095235	19,953,628
GGB132637 SGB181770	2.568736168	0.316167447	1,402,004
*Pseudomonas* SGB60145	2.383663945	0.293388278	1,430,859
*Bosea* sp. RAC05	0.720550638	0.088687526	450,027
*Acidovorax* sp. NB1	0.640593609	0.078846285	399,499
*Sphingorhabdus* SGB89971	0.071254072	0.008770026	404,413
*Flavobacterium aquatile*	0.056495918	0.006953836	87,847
*Hydrogenophaga* sp. IBVHS2	0.047935123	0.005899808	25,590
*Niveispirillum lacus*	0.040000727	0.004923568	27,190
*Erythrobacter ramosus*	0.031879744	0.003923768	82,946
GGB122063 SGB1213	0.01330544	0.001637652	25,069
*Flavobacterium chungbukense*	0.011648368	0.00143368	32,296
*Escherichia coli*	0.008383093	0.001031623	24,222
*Falsiroseomonas stagni*	0.006468354	0.000796188	44,610
*Methylophilus* SGB64807	0.002287914	0.000281792	9,965
*Aureimonas glaciei*	0.001239472	0.000152368	19,635
*Emticicia aquatilis*	0.00026211	3.22022E-05	45,583

^*a*^ -, not applicable; coverage was not calculated for the UNCLASSIFIED fraction.

**TABLE 2:** Summary of assembly statistics and genomic features for the three reconstructed circular genomes

Organism	Dolichospermum flosaquae FBCC-A233	Sphingorhabdus sp.	Brevundimonas sp.
Domain		Bacteria	
GTDB-Tk classification	d__Bacteria;p__Cyanob acteriota;c__Cyanobacte riia;o__Cyanobacteriale s;f__Nostocaceae;g__D olichospermum;s__Doli chospermum flosaquae	d__Bacteria;p__Pseudo monadota;c__Alphapro teobacteria;o__Sphingo monadales;f__Sphingo monadaceae;g__Sphing orhabdus_B;s__Sphing orhabdus_B sp023910635	d__Bacteria;p__Pseudo monadota;c__Alphapro teobacteria;o__Cauloba cterales;f__Caulobacter aceae;g__Brevundimon as;s__Brevundimonas sp040380105
Genome size (bp)	4,477,099	2,721,997	2,784,445
GC content (%)	37.09	55.6	66.83
Number of genomes		1 circular	
CDS	3,975	2,637	2,810
rRNA	15	3	3
tRNA	44	42	47
BUSCO completeness (%)	96.4	98.4	92.5
CheckM2 completeness (%)	99.98	100	99.95
CheckM2 contamination (%)	0.07	0.03	1.45

To complement the marker-based community profile and evaluate genome-wide support for the recovered contigs, trimmed short reads were mapped to the final polished genome sequences. The cyanobacterial, *Sphingorhabdus*-affiliated, and *Brevundimonas*-affiliated genomes were supported by 28,496,886, 7,869,281, and 2,954,943 mapped reads, respectively, corresponding to 51.75%, 14.29%, and 5.37% of trimmed short reads. Mean depths were 571.71×, 260.88×, and 81.06×, with ≥1× breadths of coverage of 99.48%, 99.90%, and 99.59%, respectively. Although the *Brevundimonas*-affiliated genome was not clearly resolved in the marker-based community profile, it was recovered as one of the three complete circular genomes by metagenome-resolved assembly and was supported by high genome-wide depth and breadth of coverage. These genome-wide mapping results provide coverage-based support for the three recovered genomes. Genome completeness and contamination were then evaluated using BUSCO and CheckM2. BUSCO analysis indicated completeness values of 96.40% for the cyanobacterial genome using the Nostocales data set, and 98.4% and 92.5% for the *Sphingorhabdus-* and *Brevundimonas-*affiliated genomes, respectively, using the Alphaproteobacteria data set. CheckM2 further supported the quality of the assemblies, estimating completeness/contamination values of 99.98%/0.07%, 100%/0.03%, and 99.95%/1.45% for the cyanobacterial, *Sphingorhabdus-*, and *Brevundimonas-*affiliated genomes, respectively. Together, these results indicate that the three genomes are high-quality reconstructions suitable for downstream taxonomic and functional analyses.

### Genome-based taxonomic placement and species-cluster assignment of the three reconstructed genomes

To support genome-based taxonomic placement, we compiled comparative genome data sets for the ADA group and for the alphaproteobacterial genera *Sphingorhabdus* and *Brevundimonas*. Although the initial similarity search identified *Aphanizomenon flos-aquae* as the closest reference genome, we included the broader ADA group in downstream analyses to account for the high genome-level similarity and taxonomic proximity among members of this clade (40, 41). Reference genomes for the ADA group, *Sphingorhabdus*, and *Brevundimonas* were collected from the NCBI RefSeq database (Table S1). After CheckM2-based quality filtering and FastANI screening, the final close-reference comparison set comprised 54 ADA genomes, 14 *Sphingorhabdus* genomes, and 51 *Brevundimonas* genomes for ANIb and dDDH analyses.

The screened reference sets were then used for ANIb and dDDH analyses, interpreted using commonly applied conspecific thresholds of 95%–96% ANI and 70% dDDH (42) (Table S2). For the cyanobacterial genome, ANIb and dDDH values exceeding 99% and 93%, respectively, were observed relative to multiple reference genomes annotated as *Aphanizomenon*. For the *Sphingorhabdus* genome, the closest reference showed an ANIb of 97.39% and a dDDH of 78.1, consistent with species-level relatedness. In contrast, for the *Brevundimonas* genome, the highest ANIb was 93.05% and the corresponding dDDH was 51.7% relative to the closest reference genome, remaining below commonly applied species thresholds. To place all three genomes within a consistent genome taxonomy, we further analyzed them using GTDB-Tk. In the GTDB framework, the cyanobacterial genome was assigned to the species cluster s__Dolichospermum flosaquae, with an ANI of 99.31% and an alignment fraction of 0.947 relative to the representative genome GCF_014698755.1. The *Sphingorhabdus* genome was assigned to s__Sphingorhabdus_B sp023910635, with an ANI of 95.51% and an alignment fraction of 0.743 relative to GCF_023910635.1. The *Brevundimonas* genome was assigned to s__Brevundimonas sp040380105, with an ANI of 96.51% and an alignment fraction of 0.900 relative to GCA_040380105.1.

We interpreted the RefSeq-based ANIb/dDDH comparisons and GTDB-Tk classifications as complementary but distinct taxonomic frameworks. For the cyanobacterial genome, the RefSeq-based genome comparisons showed the highest similarity to reference genomes deposited as *Aphanizomenon flos-aquae*. In the curated ADA clade comparison, ANIb and dDDH values exceeding 99% and 93%, respectively, were observed relative to multiple RefSeq genomes annotated as *Aphanizomenon*. However, the GTDB species cluster s__Dolichospermum flosaquae itself includes genomes deposited under heterogeneous organism labels, including *Aphanizomenon flos-aquae*, *Aphanizomenon* sp., *Anabaena* sp., *Dolichospermum* sp., and higher-rank cyanobacterial labels. Thus, the high RefSeq-based similarity to *Aphanizomenon*-labeled genomes reflects the taxonomic complexity of the ADA clade rather than a contradiction of the GTDB-Tk placement. Consistent with the GTDB genome-taxonomic framework, GTDB-Tk placed FBCC-A233 within s__Dolichospermum flosaquae, with 99.31% ANI. We, therefore, refer to this genome as *D. flosaquae* FBCC-A233 in genome-based taxonomic contexts. For the *Sphingorhabdus* genome, GTDB-Tk assigned the genome to s__Sphingorhabdus_B sp023910635; the suffix “_B” reflects GTDB-specific genus-level circumscription and is used here only when reporting GTDB-Tk output. In the main text, we refer to this genome as *Sphingorhabdus* sp. following validly published genus-level nomenclature. For the *Brevundimonas* genome, the highest RefSeq-based ANIb and dDDH values were 93.05% and 51.7%, respectively, below commonly applied species thresholds. Although GTDB-Tk assigned this genome to s__Brevundimonas sp040380105, we do not treat this as a formally named species assignment. Instead, we refer to it as *Brevundimonas* sp., representing a member of a GTDB-defined unnamed species cluster and a putative undescribed species-level lineage relative to currently named RefSeq species. Based on this integrated evidence, we hereafter refer to the reconstructed genomes as *Dolichospermum flosaquae* FBCC-A233, *Sphingorhabdus* sp., and *Brevundimonas* sp., respectively.

### Comparative genome architecture and genome-inferred metabolic potential across the three genomes

To assess genome architecture and genome-associated plasticity of the two associated bacteria, *Sphingorhabdus* sp. and *Brevundimonas* sp., their genomes were compared against QC-filtered genus-level reference distributions comprising 62 *Sphingorhabdus* and 416 *Brevundimonas* genomes, respectively. *Sphingorhabdus* sp. showed genome size and predicted gene content within the range of the reference distribution. In contrast, *Brevundimonas* sp. fell within the lower 10.3% and 15.9% of the *Brevundimonas* reference distribution for genome size and predicted gene count, respectively (Table 3). We then evaluated additional genome architecture and genome plasticity metrics. *Brevundimonas* sp. showed a coding density of 89.56%, corresponding to the 37.5th percentile of the *Brevundimonas* reference distribution, and a median intergenic-region length of 86 bp, corresponding to the 85.3rd percentile. *Sphingorhabdus* sp. showed a coding density of 91.54% and a median intergenic-region length of 76 bp, corresponding to the 59.7th and 82.3rd percentiles of the *Sphingorhabdus* reference distribution, respectively. No Prokka-derived pseudogene candidates were detected in either associated bacterial genome. However, IS/transposase-related CDSs were elevated in both genomes, with 32 genes in *Brevundimonas* sp. and 71 genes in *Sphingorhabdus* sp., corresponding to 11.49 and 26.08 genes/Mb, respectively.

**TABLE 3:** Genome architecture metrics of the two associated bacterial genomes relative to QC-filtered genus-level reference distributions^*a*^

	*Brevundimonas* sp.	Percentile within *Brevundimonas* references	*Sphingorhabdus* sp.	Percentile within *Sphingorhabdus* references
Reference distribution size	416 genomes	-	62 genomes	-
Genome size (Mb)	2.784	10.3	2.722	64.5
Predicted CDS count	2,810	15.9	2,637	66.1
GC content (%)	66.83	24	55.60	45.2
Coding density (%)	89.56	37.5	91.54	59.7
Median intergenic-region length (bp)	86	85.3	76	82.3
Pseudogene candidates	0	-	0	-
IS/transposase-related CDS burden	32 CDSs; 11.49 CDSs/Mb	97.6	71 CDSs; 26.08 CDSs/Mb	98.4

^*a*^ Percentile ranks indicate the position of each reconstructed genome within the corresponding genus-level reference distribution.

**Fig 2:**
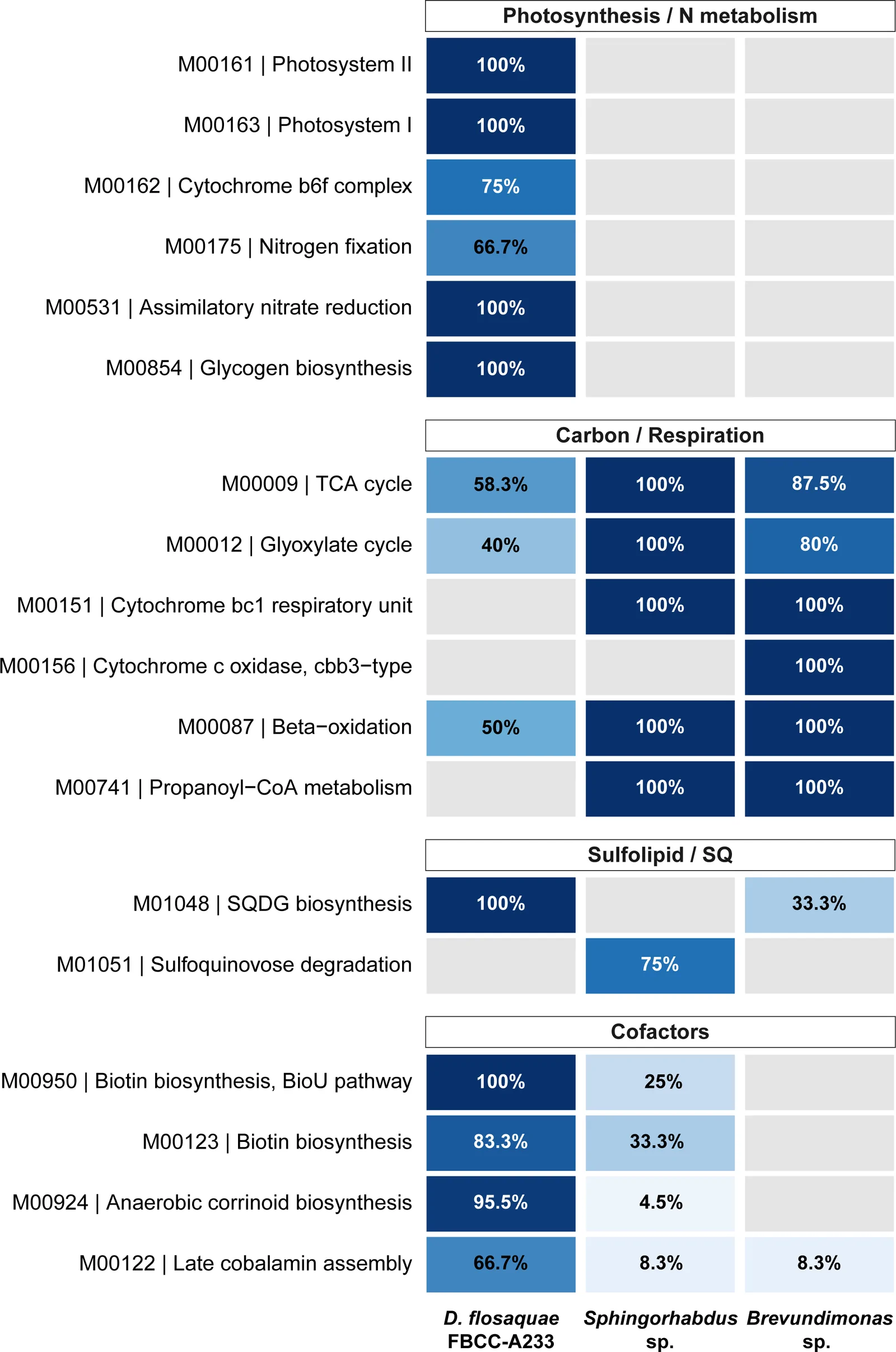
Genome-inferred metabolic potential and cofactor-related pathway asymmetry across the three reconstructed genomes. Heatmap showing selected weighted KEGG module completeness values calculated from KofamKOALA-derived KO profiles using kegg-pathway-completeness. Rows indicate selected modules related to photosynthesis and nitrogen metabolism, carbon and respiratory metabolism, sulfolipid/sulfoquinovose metabolism, and cofactor-related pathways. Color intensity represents weighted module completeness on a 0%–100% scale; gray cells indicate modules not detected in the pathway-completeness output.

To further characterize predicted metabolic differences among the three genomes, selected KEGG module completeness was recalculated (Fig. 2; Table S3). These module profiles were interpreted as genome-inferred metabolic potential. *D. flosaquae* FBCC-A233 showed high completeness for photosynthesis- and nitrogen-related modules, including photosystem I, photosystem II, assimilatory nitrate reduction, and glycogen biosynthesis. In contrast, the two associated bacterial genomes showed higher completeness for several carbon and respiratory modules, including the TCA cycle, glyoxylate cycle, cytochrome bc1 respiratory unit, β-oxidation, and propanoyl-CoA metabolism. *Brevundimonas* sp. additionally showed completeness for the cbb3-type cytochrome *c* oxidase module. Because the glyoxylate cycle and respiratory-chain modules are common bacterial functions, these profiles were interpreted as general heterotrophic and respiratory potential rather than direct evidence that the associated bacteria utilize cyanobacterial organic matter or navigate phycosphere nutrient gradients. A more specific substrate-linked asymmetry was observed for sulfolipid-related modules. SQDG biosynthesis was detected primarily in *D. flosaquae* FBCC-A233, whereas sulfoquinovose degradation was detected in *Sphingorhabdus* sp. This paired distribution suggests a genome-inferred sulfolipid/sulfoquinovose metabolic complementarity but does not directly demonstrate sulfolipid-derived carbon transfer. Cofactor-related modules showed marked asymmetry. The BioU-mediated biotin biosynthesis module was complete in *D. flosaquae* FBCC-A233, partial in *Sphingorhabdus* sp., and not detected in *Brevundimonas* sp. Similarly, the anaerobic corrinoid biosynthesis module was highly complete in *D. flosaquae* FBCC-A233 but low or not detected in the associated bacterial genomes. In contrast, late cobalamin assembly was incomplete under the weighted module criteria, indicating that cobamide-related interpretations require gene-level evaluation.

**Fig 3:**
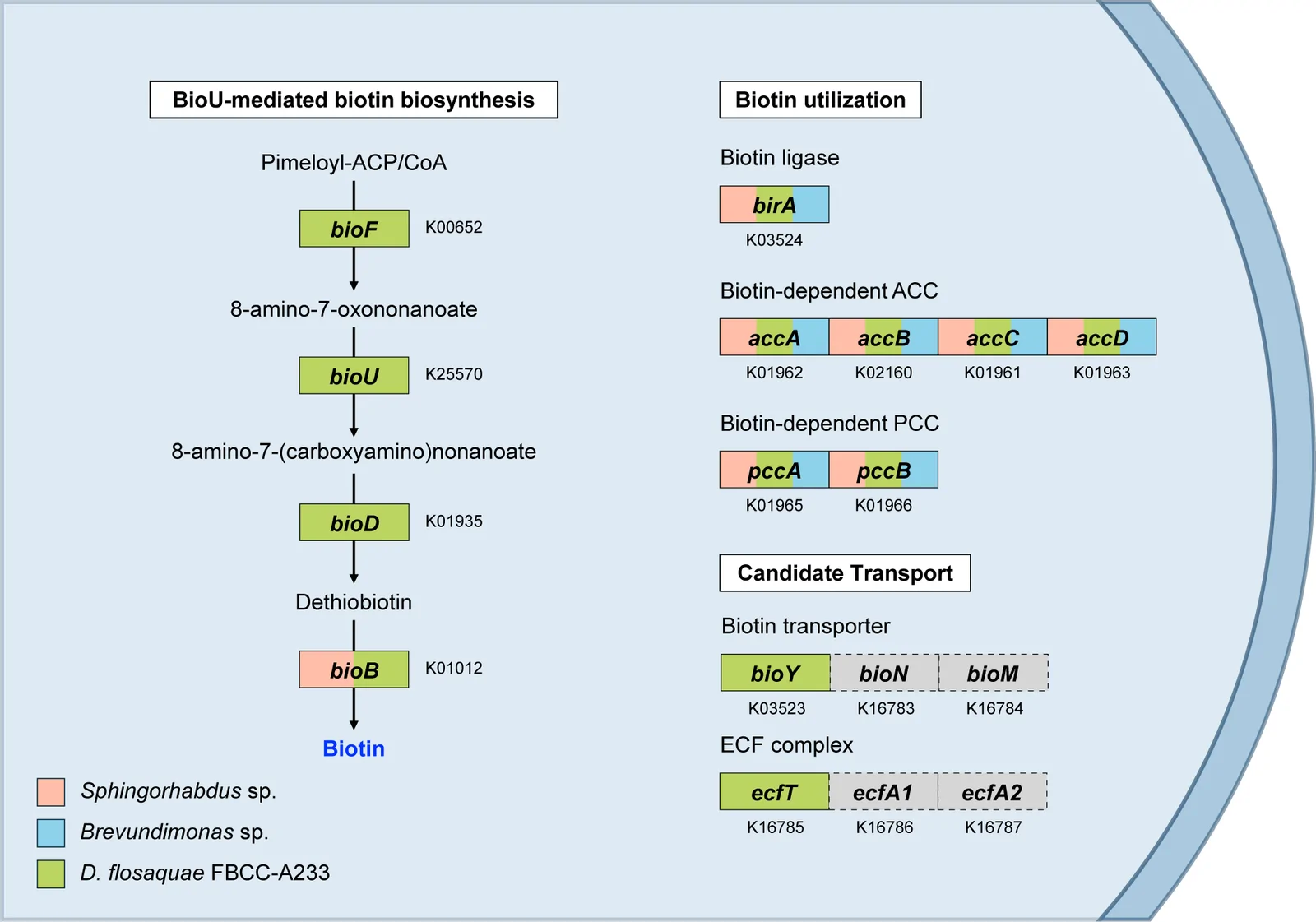
KO-level reconstruction of biotin biosynthesis, utilization, and candidate transport. Gene boxes indicate threshold-passing KofamKOALA hits in *D. flosaquae* FBCC-A233, *Sphingorhabdus* sp., and *Brevundimonas* sp. FBCC-A233 encoded a BioU-mediated *de novo* biotin biosynthesis route, whereas the associated bacterial genomes lacked complete *de novo* biosynthesis. All three genomes encoded *birA* and *accABCD*, and the associated bacteria additionally encoded *pccAB*, indicating biotin-dependent carboxylase potential. Candidate biotin uptake genes were not detected in the associated bacterial genomes; therefore, this panel represents genome-inferred biotin-related asymmetry rather than demonstrated biotin exchange. Dashed gray boxes indicate genes that were not detected in the corresponding genome.

We reconstructed cofactor-related pathways at the KO level, focusing on biotin and cobamide metabolism. The biotin pathway showed a clear genome-inferred asymmetry among the three genomes (Fig. 3). *D. flosaquae* FBCC-A233 encoded a BioU-mediated *de novo* biotin biosynthesis route from pimeloyl-ACP/CoA to biotin, including *bioF*, *bioU*, *bioD*, and *bioB*, whereas the canonical *bioA* route was not detected. In contrast, *Sphingorhabdus* sp. retained only the terminal *bioB* step, and *Brevundimonas* sp. lacked the core *de novo* biotin biosynthesis genes. All three genomes encoded *birA* and *accABCD*, indicating conserved biotin-utilization machinery. In addition, *Sphingorhabdus* sp. and *Brevundimonas* sp. encoded *pccAB*, suggesting additional biotin-dependent propionyl-CoA carboxylase activity and potential biotin demand beyond acetyl-CoA carboxylase. However, candidate biotin transport genes were not detected in the associated bacterial genomes, and no complete BioMNY or ECF-type biotin transporter was reconstructed.

**Fig 4:**
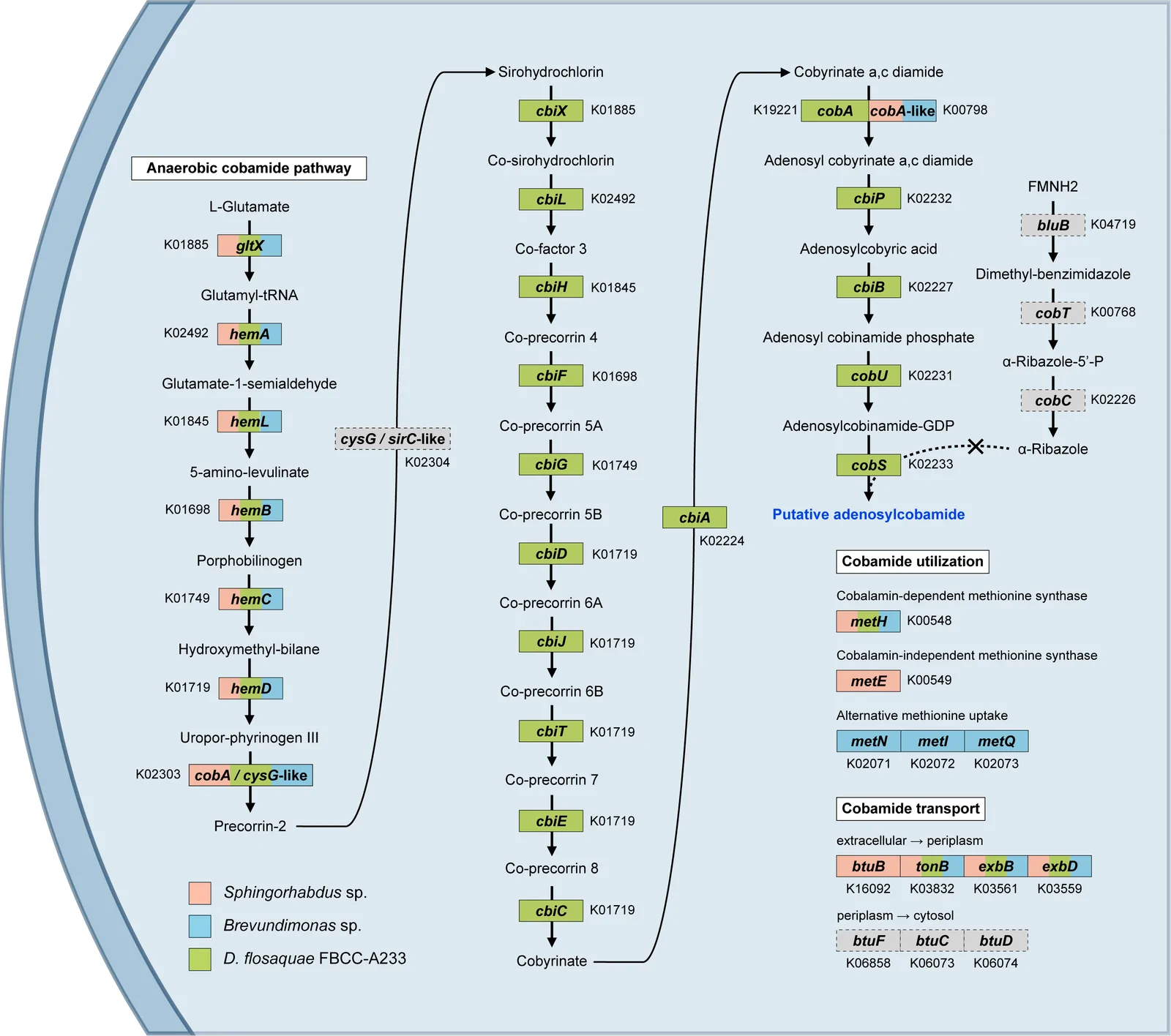
KO-level reconstruction of cobamide/corrinoid-related metabolism. Metabolite-to-metabolite map showing selected steps from tetrapyrrole precursor synthesis to anaerobic corrinoid-ring biosynthesis, late cobamide assembly, canonical DMB lower-ligand formation, and cobamide-related utilization. Gene boxes indicate threshold-passing KofamKOALA hits in *D. flosaquae* FBCC-A233, *Sphingorhabdus* sp., and *Brevundimonas* sp. *D. flosaquae* FBCC-A233 encoded most anaerobic corrinoid-ring biosynthesis genes, whereas the sirohydrochlorin-forming step and canonical DMB lower-ligand genes (*bluB*, *cobT*, and *cobC*) were not detected.

For cobamide metabolism, *D. flosaquae* FBCC-A233 encoded extensive anaerobic corrinoid biosynthesis potential (Fig. 4). The reconstructed pathway connected glutamyl-tRNA-derived tetrapyrrole precursors to uroporphyrinogen III through *gltX*, *hemA*, *hemL*, *hemB*, *hemC*, and *hemD*, followed by the *cobA*/*cysG*-like step from uroporphyrinogen III to precorrin-2. However, the precorrin-2 dehydrogenase/sirohydrochlorin-forming step could not be confidently assigned. Downstream of sirohydrochlorin, *D. flosaquae* FBCC-A233 retained most genes for anaerobic corrinoid-ring biosynthesis, supporting genome-inferred formation of cobyrinate a,c-diamide-related intermediates. *D. flosaquae* FBCC-A233 also encoded late cobamide assembly genes, including *cobA*, *cbiP*, *cbiB*, *cobU*, and *cobS*, consistent with potential conversion toward adenosylcobinamide-GDP and a putative adenosylcobamide. However, canonical DMB-associated lower-ligand genes were not detected, including *bluB*, which mediates FMNH₂-to-DMB conversion, *cobT*, which converts *DMB* to α-ribazole-5′-phosphate, and *cobC*, which converts α-ribazole-5′-phosphate to α-ribazole. Therefore, canonical DMB-containing cobalamin biosynthesis could not be inferred, and the final cobamide identity remains unresolved. Cobamide-related utilization and transport profiles were also asymmetric. *D. flosaquae* FBCC-A233 encoded the cobamide-dependent methionine synthase *metH* but lacked the cobalamin-independent alternative *metE*. In the associated bacterial genomes, genes for *de novo* cobamide biosynthesis were largely absent. *Sphingorhabdus* sp. encoded both *metH* and *metE*, whereas *Brevundimonas* sp. encoded only *metH. Brevundimonas* sp. also encoded the methionine ABC transporter genes *metN*, *metI*, and *metQ*, suggesting a possible alternative route for methionine acquisition. Regarding cobamide transport, *btuB* was identified in *Sphingorhabdus* sp., together with the general TonB energy-associated components *tonB*, *exbB*, and *exbD*; however, the complete canonical cobalamin transporter complex *btuFCD* was not detected in any of the three genomes.

## DISCUSSION

In this study, we reconstructed three complete circular genomes from a unialgal xenic culture, representing the cyanobacterium and two associated bacteria, and used them to examine genome-resolved functional potential. The recovery of complete circular genomes enabled direct pathway-level comparisons that would not have been possible with 16S rRNA gene profiling alone or with fragmented genome assemblies. However, non-axenic cyanobacterial cultures can retain co-maintained heterotrophic bacteria and may serve as tractable systems for generating experimentally testable hypotheses about cyanobacteria–bacteria associations (43, 44). Because culture-associated communities may differ from natural bloom communities and can be shaped by cyanobacterial genotype, culture history, physiology, and cultivation conditions, the bacterial genomes recovered here should be interpreted as culture-associated members of *D. flosaquae* FBCC-A233 rather than as a complete representation of the original environmental phycosphere community.

The cyanobacterial genome showed the highest similarity to *Aphanizomenon flos-aquae* reference genomes, whereas GTDB-Tk placed it within the s__Dolichospermum flosaquae species cluster. This discrepancy reflects longstanding taxonomic challenges within the ADA group, where high genomic similarity and morphological plasticity have repeatedly confounded genus-level boundaries (41, 45). This result underscores the importance of genome-based frameworks for taxonomic placement within the ADA group. The two associated bacterial genomes were assigned to the alphaproteobacterial genera *Sphingorhabdus* sp. and *Brevundimonas* sp. Members of these genera, particularly *Brevundimonas* sp., have frequently been reported from cyanobacterial blooms and phycosphere-associated communities (12, 15, 46), where heterotrophic bacteria may benefit from cyanobacterial exudates and other organic compounds (47). However, the genus-level reference distributions used here were not curated as ecology-matched phycosphere-associated, symbiotic, or free-living control groups. Therefore, genome architecture comparisons should be interpreted as comparisons against heterogeneous genus-level backgrounds rather than as direct evidence of phycosphere-specific or symbiosis-associated genome evolution.

The genome architecture metrics calculated here do not support a strong conclusion of classical genome streamlining. Although the *Brevundimonas* sp. genome was smaller and contained fewer predicted genes relative to the genus-level reference distribution, its coding density was not elevated and its median intergenic-region length was not reduced. In addition, both associated bacterial genomes showed elevated IS/transposase-related gene burden, whereas no Prokka-derived pseudogene candidates were detected. These results suggest reduced genome size in *Brevundimonas* sp. relative to the genus-level background, but they do not establish genome streamlining as a definitive evolutionary process. We, therefore, interpret the pattern more conservatively as reduced genome size and gene content, accompanied by elevated mobile-element-related gene burden, rather than as clear evidence of classical genome streamlining.

The module completeness profiles indicated genome-inferred metabolic asymmetry among the three genomes, with photosynthesis- and nitrogen-related modules concentrated in *D. flosaquae* FBCC-A233 and general heterotrophic and respiratory modules more complete in the associated bacterial genomes. Because these bacterial modules are widespread functions, we interpret them as predicted metabolic potential rather than direct evidence of cyanobacterium-derived substrate use. The distribution of cofactor-related genes suggests cofactor-related metabolic asymmetry, but not confirmed biotin cross-feeding. *D. flosaquae* FBCC-A233 was the only genome encoding the BioU-mediated *de novo* biotin biosynthesis route, whereas the associated bacterial genomes lacked complete *de novo* biosynthetic pathways. *Sphingorhabdus* sp. retained only *bioB*, suggesting that it may complete the terminal conversion to biotin only if late-stage precursors such as dethiobiotin are available. *Brevundimonas* sp. lacked the core *de novo* biotin biosynthesis genes. Nevertheless, both associated bacterial genomes retained biotin-utilization machinery, including *birA*, *accABCD*, and *pccAB*, indicating potential biotin-dependent carboxylase activity. The inclusion of *pccAB* strengthens the interpretation that the associated bacteria may have biotin demand beyond acetyl-CoA carboxylase. However, candidate biotin uptake genes were not detected in the associated bacterial genomes, and biotin export, salvage, and growth dependence were not experimentally tested. Thus, *D. flosaquae* FBCC-A233 should be viewed as a candidate biotin producer, whereas biotin acquisition by the associated bacteria remains unresolved. A similar asymmetry was observed in cobamide-related metabolism, but this pattern should be interpreted as genome-inferred potential rather than confirmed cobamide production or exchange. *D. flosaquae* FBCC-A233 encoded most genes required for anaerobic corrinoid-ring biosynthesis, including the *cbi* genes connecting sirohydrochlorin to cobalt-precorrin intermediates and cobyrinate a,c-diamide. This supports *D. flosaquae* FBCC-A233 as a candidate corrinoid producer within the culture. However, the pathway reconstruction also revealed important gaps. The precorrin-2 dehydrogenase/sirohydrochlorin-forming step could not be confidently assigned, and the canonical DMB lower-ligand branch was incomplete because *bluB*, *cobT*, and *cobC* were not detected. Therefore, although the gene profile is compatible with non-canonical cobamide biosynthesis, the actual cobamide structure was not determined in this study and requires metabolite-level validation. The associated bacterial genomes lacked complete *de novo* cobamide biosynthesis but retained cobamide-related utilization genes. The distribution of methionine synthase genes suggests possible differences in cobamide-related dependence: *D. flosaquae* FBCC-A233 and *Brevundimonas* sp. encoded *metH* but lacked *metE*, whereas *Sphingorhabdus* sp. encoded both *metH* and *metE*, potentially providing greater flexibility under cobamide-limited conditions. However, these profiles do not demonstrate cobamide cross-feeding. Although *Sphingorhabdus* sp. encoded a candidate outer-membrane B12 receptor (*btuB*) and general TonB-associated components, the complete canonical *btuFCD* transporter was not detected in any of the three genomes. In *Brevundimonas* sp., the co-occurrence of *metH* with *metN*, *metI*, and *metQ* suggests that methionine uptake may provide an alternative route to buffer methionine demand, but it does not establish cobamide uptake. Thus, cobamide identity, transport, remodeling, and exchange remain unresolved and should be tested using targeted cobamide profiling, supplementation assays, and transporter validation.

Several limitations should nevertheless be acknowledged. First, the genomes were reconstructed from a culture collection-maintained unialgal xenic culture rather than directly from an environmental sample. Repeated single-filament isolation during culture maintenance may reduce loosely associated microorganisms and favor bacterial members that remain attached to cyanobacterial filaments, cell surfaces, or mucilaginous material. However, because we did not perform longitudinal community profiling across passages, axenic culture comparisons, or microscopy-based localization of bacterial cells, the recovered bacterial genomes should be interpreted as culture-associated or co-maintained bacterial members of *D. flosaquae* FBCC-A233, rather than experimentally confirmed symbionts or a complete representation of the original environmental phycosphere community. Second, the marker-based community profile and genome-wide read-mapping validation should be interpreted as complementary but non-equivalent analyses. The marker-based profile provides an estimate of community composition based on clade-specific marker genes and can be influenced by unclassified reads, reference-database representation, and strain-level divergence. By contrast, read mapping to the final polished genome sequences evaluates whether the recovered genomes are supported across their full length. Thus, mapping depth and breadth provide assembly-support evidence for the three reconstructed genomes, but should not be interpreted as a replacement for whole-community taxonomic profiling. This distinction may explain why the marker-based community profile did not fully mirror the three recovered genomes, including the weak or absent marker-based detection of some recovered taxa. Low-abundance or marker-divergent community members not represented among the final assemblies may, therefore, still have been present in the unialgal xenic culture. Third, because the present interpretations are based on genome annotation, gene presence does not necessarily indicate gene expression or metabolite production. In addition, transporter-level assignments and cofactor exchange mechanisms remain incompletely resolved. Future studies integrating supplementation assays, transcriptomic profiling, and metabolic quantification will be important for clarifying the physiological dynamics of the *Dolichospermum*-associated consortium.

## Data Availability

The raw sequencing reads and genome assemblies generated in this study have been deposited under NCBI BioProject PRJNA1404972. Raw Illumina and Nanopore reads are available in the NCBI SRA under SRR38145386 and SRR38145385, respectively. The BioSample accessions are SAMN54770858 for *D. flosaquae* FBCC-A233, SAMN54770859 for *Sphingorhabdus* sp., and SAMN54770860 for *Brevundimonas* sp.

## SUPPLEMENTAL MATERIAL

Supplemental legends spectrum.01014-26-s0001.docx Legends for Tables S1 to S4.

Table S1 spectrum.01014-26-s0002.xlsx Reference genome metadata and quality-control summary.

Table S2 spectrum.01014-26-s0003.xlsx Genome relatedness analysis of reconstructed genomes.

Table S3 spectrum.01014-26-s0004.xlsx Genome-specific KEGG module completeness profiles.

Table S4 spectrum.01014-26-s0005.xlsx KO-level reconstruction of cofactor-related genes.

ASM does not own the copyrights to Supplemental Material that may be linked to, or accessed through, an article. The authors have granted ASM a non-exclusive, world-wide license to publish the Supplemental Material files. Please contact the corresponding author directly for reuse.
