# Supplementary material for: Complete genomes from a xenic Dolichospermum flosaquae FBCC-A233 culture reveal genome-inferred metabolic asymmetry with associated bacteria
Source: Microbiol Spectr. 2026 Jul 31;14(9):e01014-26. doi: 10.1128/spectrum.01014-26 (PMC13532358; doi:10.1128/spectrum.01014-26)
Supplement: Supplementary file 1 [file spectrum.01014-26-s0001.docx]

Table S1. Reference genome metadata and quality-control summary

Table S2. Genome relatedness analysis of reconstructed genomes

Table S3. Genome-specific KEGG module completeness profiles

Table S4. KO-level reconstruction of cofactor-related genes
